# Model Development and Exergy Analysis of a Microreactor for the Steam Methane Reforming Process in a CFD Environment

**DOI:** 10.3390/e21040399

**Published:** 2019-04-15

**Authors:** Zia ur Rahman, Iftikhar Ahmad, Manabu Kano, Jawad Mustafa

**Affiliations:** 1 Department of Chemical Engineering, National University of Sciences and Technology (NUST), H-12 Islamabad 46000, Pakistan; 2 Department of Systems Science, Graduate School of Informatics, Kyoto University, Yoshida-Honmachi, Sakyo-Ku, Kyoto 606-8501, Japan; 3 Department of Chemical and Petroleum Engineering, College of Engineering, United Arab Emirates University, Al-Ain 73000, UAE

**Keywords:** steam methane reforming, computational fluid dynamics, monolith reactor, physical exergy, chemical exergy, CHEMKIN, rhodium catalyst, simple algorithm

## Abstract

Steam methane reforming (SMR) is a dominant technology for hydrogen production. For the highly energy-efficient operation, robust energy analysis is crucial. In particular, exergy analysis has received the attention of researchers due to its advantage over the conventional energy analysis. In this work, an exergy analysis based on the computational fluid dynamics (CFD)-based method was applied to a monolith microreactor of SMR. Initially, a CFD model of SMR was developed using literature data. Then, the design and operating conditions of the microreactor were optimized based on the developed CFD model to achieve higher conversion efficiency and shorter length. Exergy analysis of the optimized microreactor was performed using the custom field function (CFF) integrated with the CFD environment. The optimized catalytic monolith microreactor of SMR achieved higher conversion efficiency at a smaller consumption of energy, catalyst, and material of construction than the reactor reported in the literature. The exergy analysis algorithm helped in evaluating length-wise profiles of all three types of exergy, namely, physical exergy, chemical exergy, and mixing exergy, in the microreactor.

## 1. Introduction

The demand for hydrogen production is rapidly increasing due to its immense importance in the petroleum and chemical industry. Hydrogen is mainly used for the up-gradation of fossil fuels and ammonia production. The use of hydrogen as a fuel has also recently increased due to its high heating value and less-polluting nature [1]. There are a variety of other applications of hydrogen such as fuel cells, metal production, and power generation [2].

The steam reforming of methane gas is the most abundantly used process for the production of hydrogen. Saeidi et al. estimated that 48% of all hydrogen production came from the steam reforming of natural gas [3]. However, steam methane reforming (SMR) has several limitations, such as heat transfer limitation, mass transfer limitation, and catalyst deactivation [1]. To cope with these limitations, conventional SMR is being transformed into micro-reforming technology, which overcomes the heat transfer limitation and enhances the mass transfer; as a result, micro-reforming technology improves the conversion efficiency [4]. The intensified form of SMR (i.e., micro size plant) increases profit margins above 70% for the same capacity of H_2_ production [1].

Granlund et al. revealed that a microreactor having multiple air inlets produces a high yield of hydrogen as compared to the conventional monolith reactor by using computational fluid dynamics (CFD) [5]. Bhat et al. presented a review on various research directions in the area of process intensification of the SMR process [6]. Chen et al. developed the CFD-based model of a catalytic microcombustor and evaluated the combustion characteristics and stability of a methane–air mixture [7]. Yu et al. developed a microreactor for the steam reforming of methanol and analyzed the performance of the microreactor with optimum catalyst coating under different operating conditions [8]. Theampetch, A. et al. produced a hybrid system of SMR which transformed some part of methane to hydrogen by using the heat of exhaust gases of an internal combustion engine, and they showed through CFD simulation that the homogeneity of temperature in the longitudinal and radial directions was indispensable for high methane conversion efficiency [9]. Kansha, Y. et al. compared the energy requirement for self-heat recuperative systems and conventional thermal processes. They concluded that the recuperative thermal processes had a higher capability to save energy than the conventional thermal processes in the industries [10]. Hosseini and Wahid investigated the conventional and flameless combustion in a lab-scale furnace based on exergy analysis and concluded that the major irreversibilities were caused by the high temperature gradient present in the reactor chamber [11]. 

An et al. investigated different microreactor structures, such as parallel, oblique pin, pinhole, wavy, and coil, with CFD simulation to achieve a high-performance configuration with respect to heat transfer, reaction rates, and their flow characteristics. They concluded that the pinhole configuration could achieve better performance than the other structures [12]. Kashid et al. developed the CFD model of a slug flow microreactor to examine the impact of viscosity on the fluid flow streamlines within the slugs. They summarized that the variation in viscosity had no effect on the flow patterns within the slugs [13]. 

Since SMR is endothermic and energy intensive, a robust energy analysis of the micro-scale SMR is also needed [14]. The concept of exergy has attracted the attention of researchers and process designers because of its capability to evaluate the true thermodynamic potential of a process. Exergy analysis helps in the identification of the locations, causes, and true magnitude of irreversibility and losses in a system [15]. Exergy analysis of the process models in the CFD environment has been reported in the literature.

Boulenouar and Ouadha performed a CFD-based exergy analysis of flow in a supersonic steam ejector and found that the main source of irreversibility was the nozzle due to high levels of pressure and velocity gradients [16]. Mustafa et al. developed a CFD-based model for exergy analysis of naphtha reforming reactors and concluded that the total exergy of the stream increased along the reactor [17]. Alabi et al. compared experimental and CFD-based exergy analysis procedures for the design optimization of the airframe subsystem of aircraft. They observed that exergy-based analysis has a significant advantage over an energy-based approach [18]. Gunjo et al. developed a CFD model for a flat plate solar collector to perform exergy and energy analysis. It was observed that the performance of the collector was affected by inlet water temperature, ambient temperature, solar insolation, mass flow rate, working fluids, insulation thickness, and collector heat loss factor [19]. 

Debnath et al. performed CFD-based exergy efficiency analysis of air hydrogen detonation in a pulse detonation combustor. They concluded that the deflagration combustion process had higher exergy losses than the detonation combustion process [20]. Erguvan et al. analyzed energy and exergy of unsteady cross-flow overheated circular cylinders in the CFD environment. It was found that exergy efficiency could be increased by selecting specific pitch ratios for different Reynolds numbers [21]. Alabi et al. reported the use of a CFD-based exergy analysis procedure for an aircraft system. They presented different exergy calculations and compared empirical models of the airframe subsystem to CFD models [22]. Yong-an et al. performed an exergy analysis of exhaust gas in a kitchen burning liquefied gas [23]. Farmahini-Farahani investigated different geometrical parameters for the thermal stratification of cold water tanks by CFD-based exergy analysis and concluded that decreasing the diameter of inlet/outlet reduced initial mixing within the tank and thus thermal stratification happened sooner, leading to higher exergy in the tank [24]. 

In this work, exergy analysis of a monolith microreactor of SMR was performed by using a computational fluid dynamics (CFD) model. Initially, the CFD model was developed using literature data. The monolith catalyst bed was used by considering its large surface area, low-pressure drop, high durability, and high mechanical strength. The surface-based approach was used for modeling reaction rates. To incorporate reaction kinetics, the CHEMKIN mechanism was used. Size of the reactor and operation conditions were optimized to realize higher conversion efficiency and smaller capital and operational costs. To analyze true thermodynamic efficiency, its exergy analysis was performed by developing a custom field function (CFF)-based algorithm. The exergy analysis algorithm helps in evaluating length-wise profiles of all three types of exergy, namely, physical exergy, chemical exergy, and mixing exergy, in the microreactor. 

In Section 2, the process description and concepts of exergy are discussed, followed by a description of the model development in Section 3. Section 4 describes the results and discussion, while Section 5 concludes the work.

## 2. Process and Exergy 

In this section, the SMR process and exergy concepts are described.

### 2.1. Process Description

**Figure 1:**
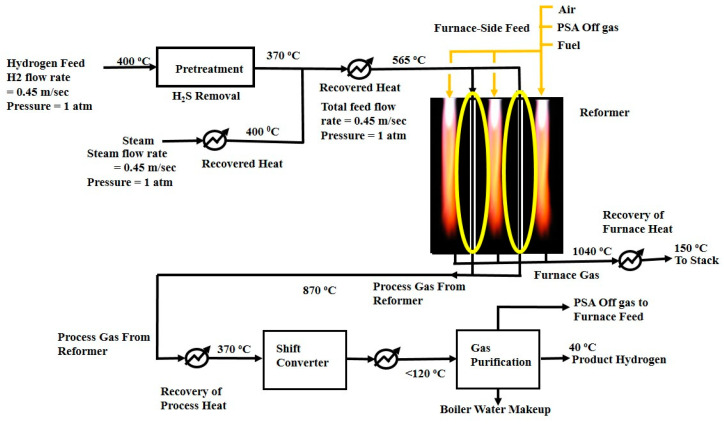
Flow diagram of the conventional steam methane reforming process [22].

Hydrogen is produced mainly in four steps—natural gas pretreatment, reforming process, water–gas shift reaction, and gas purification, as shown in Figure 1 [25]. 

In the pretreatment step, the impurities present in the natural gas feed are separated to prevent the catalyst from poisoning. The zinc oxide bed captures the sulfur-containing compounds such as hydrogen sulfide and leaves the gas with sulfur content less than 1 ppm at 335 °C [26]. 

In the reforming process, the natural gas–steam mixture is catalytically converted into hydrogen, carbon monoxide, and carbon dioxide in the reformer according to the following reactions:

CH_4_ + H_2_O ↔ 3H_2_ + CO       ∆H°_298_ = +206 kJ/mol (1)

CH_4_ + H_2_O ↔ 4H_2_ + CO_2_       ∆H°_298_ = +165 kJ/mol (2)

**Figure 2:**
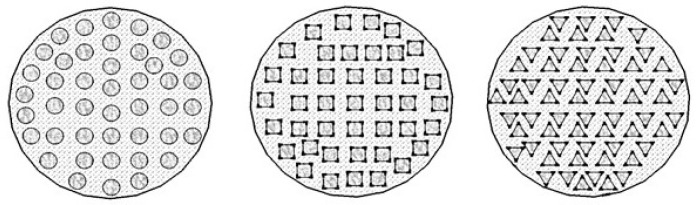
Circular, square, and trigonal shape arrangement of monolith reactors [4].

The reforming reaction is highly endothermic; therefore, heat is provided through external burners to maintain the temperature and pressure at 1100–1500 °C and 1–5 atm, respectively. The side burners are operated to heat the furnaces which supply heat to the reformer tubes through forced convection and radiation. The reformer furnace normally consists of a number of tubes (i.e., in the range of 40–400) determined by the plant design capacity. Different shapes and arrangements of the monolithic support channels are shown in Figure 2 [4].

The third step is the water–gas shift reaction. The syngas exiting the reformer takes part in a water–gas shift (WGS) reaction. It converts CO and H_2_O in the syngas to CO_2_ and H_2_ using the following reaction:

CO + H_2_O ↔ CO_2_ + H_2_       ∆H°_298_ = −41 kJ/mol (3)

In the fourth step, which is the purification of hydrogen stream, CO_2_ is removed through chemical absorption, and unreacted methane is separated by pressure swing adsorption (PSA). 

**Figure 3:**
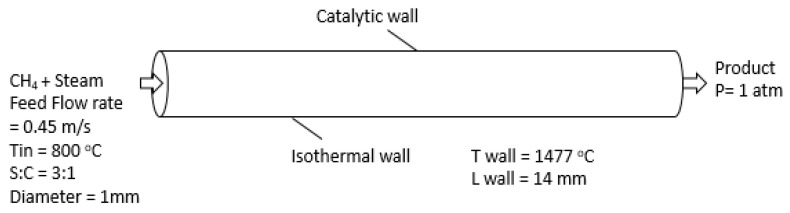
Tubular reactor model representing a single pore of a monolithic catalyst.

In this work, a two-dimensional CFD model of a single reformer tube of monolith catalytic reactor was designed [27]. The single reformer tube is shown in Figure 3, and the model parameters are mentioned in Table 1.

The reactor is fed with a mixture of methane and water vapor. Inlet velocity is maintained at 0.45 m/s and a temperature of 800 °C. The mole fraction of methane and steam are 0.23 and 0.77, respectively. A heating jacket surrounds the monolithic reactor and provides the heat of reaction to maintain a constant temperature of 1477 °C at reactor walls. The inlet H_2_O/CH_4_ molar ratio is kept constant at 3, and the operating pressure is atmospheric.

### 2.2. Exergy Concept

The term exergy was first introduced by Zoran Rant, and it means the amount of work (-erg) that is released (ex-) [28]. Exergy is defined as the maximum amount of work obtainable when an energy carrier is brought from its initial state to a state of thermodynamic and chemical equilibrium with the environment, referred to as the dead state. Exergy can be viewed as a measure of energy usefulness or quality [17]. Exergy is consumed during real processes due to irreversibilities [29]. Conventionally, exergy of a stream is classified into three parts: physical exergy, chemical exergy, and mixing exergy. A general expression of exergy is given by the following:

*E* = *E*^ph^ + *E*^ch^ + *E*^mix^ (4)

where *E* represents total molar exergy of a stream, *E*^ph^ is the molar physical exergy, *E*^ch^ is the molar chemical exergy, and *E*^mix^ is the molar mixture exergy [28].

Physical exergy represents the thermomechanical portion of the total exergy stream. It is the maximum obtainable amount of work when this stream is brought from actual conditions (*T, P*) to thermomechanical equilibrium at ambient temperature (*T_o_, P_o_*) by reversible processes. 

On a molar basis, physical exergy is given by the following:

(5) $$E^{\mathrm{ph}}=RT_{o}\sum_{i\;=\;1}^{n}ln\frac{p_{i}}{p_{o}}+\;\sum_{i\;=\;1}^{n}C_{p_{i}}^{\mathrm{mean}}\left(T_{i}-T_{o}-T_{o}\ln\left(\frac{T_{i}}{T_{o}}\right)\right)$$

(6) $$C_{p_{i}^{\mathrm{mean}}}=\;\int_{T_{1}}^{T_{2}}C_{p_{i}}dT$$

(7) $$C_{p_{i}}\left(\frac{j}{mol.K}\right)=\;a_{i}+\;b_{i}T+\;c_{i}T^{2}+d_{i}T^{3}$$

where a_i_, b_i_, c_i_, and d_i_ are heat capacity coefficients and *R* is the ideal gas constant [28]. *P_i_* and *T_i_* represent the partial pressure and temperature of individual components, respectively, at each point in the reactor [17].

Chemical exergy is the maximum obtainable work from a material stream by taking it from a state of thermomechanical equilibrium to a state of thermomechanical and chemical equilibrium with the environment [17]. Chemical exergy of a material stream on a molar basis is given by Equation (8):

(8) $$E^{\mathrm{ch}}=\overset{n}{\underset{i\;=\;1}{\sum }}\nu_{i}{\bar{G}}_{i}\left(\mathrm{Reactants}\right)-\overset{n}{\underset{i\;=\;1}{\sum }}\nu_{i}{\bar{G}}_{i}\left(\mathrm{Products}\right)$$

(9) $${\bar{G}}_{i}=\;G_{f}^{0}+\left[{\bar{G}}_{i\left(T,P\right)}-\;{\bar{G}}_{i\left(To,Po\right)}\right]$$

where $v_{i}$ is the respective stoichiometric coefficients, ${\bar{G}}_{i}$ is the molar Gibbs function of components *i*, and $G_{f}^{0}$ is the molar Gibbs function of formation at a reference temperature and pressure [30]. 

Mixing exergy accounts for the mixing effect arising from the isothermal and isobaric mixing of pure components at process conditions [31]. It can be calculated by the following:

(10) $$E_{mix}=\sum_{i\;=\;1}^{n}x_{i}T_{o}R\ln x_{i},$$

where $x_{i}$ is a mass fraction of component *i*. Mixing exergy is always a negative value because the mixing of different components decreases the exergy continuously along the length of the reactor [28]. It can also be written in the form of $\sum_{i\;=\;1}^{n}\frac{x_{i}RT_{o}\ln p_{i}}{P_{o}}\;$, where *P_i_* is the partial pressure of each component (*P_i_ =* *χ_i_ P_total_)* according to Dalton’s law [32].

## 3. Model Development

In Section 3.1, details regarding geometry and mesh preparation are provided, while in Section 3.2, boundary and cell zone conditions are described. A set of CFD-based conservative equations are provided in Section 3.3. The kinetic models describing the surface catalytic reactions are presented in Section 3.4, and numerical schemes to solve those equations are given in Section 3.5. The following assumptions are taken for model development:

- The equilibrium state is reached and the maximum yield is achieved.
- There is negligible heat loss from the reactor wall to the surroundings.
- The catalytic wall is isothermal in condition.
- The flow regime is laminar in the reaction channel, and steady-state operation is reached.
- The gas mixture is treated as an incompressible ideal gas. The density of the mixture is constant as calculated from ideal gas law.
- The gas-phase non-catalytic reaction can be neglected so that only the surface reactions are modeled.

### 3.1. Geometry and Meshing

**Figure 4:**
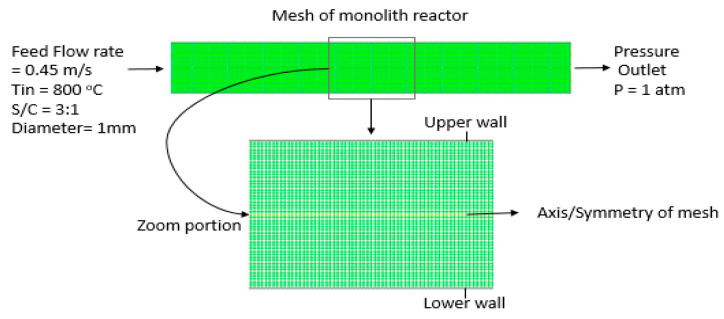
Computational grid of monolith reactor and zoom portion of the grid.

ANSYS Design Modeler® 16.0 was used to create geometry and mesh. The reactor considered in this study is cylindrical, with a height of 1 mm and length of 14 mm. The reactor consists of an isothermal catalytic (rhodium catalyst) wall as shown in Figure 3 [33]. The simulation was carried out in an axisymmetric mesh; for effective visualization, the axisymmetric mesh was mirrored around its axis. The zoomed version of the mesh is shown in Figure 4. The computational mesh consists of 7200 cells and 7525 nodes. Three important parameters to evaluate the mesh quality are minimum orthogonal quality, maximum ortho-skew, and maximum aspect ratio; in this study, they are 1.0, 0.0, and 1.4, respectively. Table 2 presents the mesh properties.

### 3.2. Boundary and Cell Zone Conditions

A single tube of monolith reactor is shown in Figure 3. Boundary conditions are defined at the inlet and outlet of the reactor. The inlet boundary condition, that is, the velocity, temperature, pressure, and the composition of inlet gas mixture, in each channel is set at uniform values. The catalytic wall of the reactor tube is kept isothermal. Each reforming tube is tightly packed with rhodium (Rh) catalyst particles. It facilitates the formation of hydrogen fuel from steam and methane through the highly endothermic SMR reactions. It also plays a role as an intermediate medium to enhance the rate of heat transfer to the tube-side gas mixture in the simulations [25]. Considering the Knudsen number of the flow, the flow regime is a continuum, and hence the no-slip condition is used on the microreactor walls. The outflow mixture is discharged to atmospheric pressure. Table 1 shows the model parameters.

### 3.3. CFD Model Describing the Flow Field

A two-dimensional steady-state model is employed by incorporating it with the detailed homogeneous and heterogeneous reaction schemes in CHEMKIN and Surface-CHEMKIN format. Steady-state continuity, momentum, energy, and species equations in the fluid phase are as follows [34].

Continuity equation

(11) $$\frac{\partial \rho }{\partial t}+\frac{\partial \left(\rho u_{x}\right)}{\partial x}+\frac{\partial \left(\rho u_{y}\right)}{\partial y}=\;S_{m}$$

where ρ, $u_{x},$ and $u_{y}$ are the density, and the velocity in *x* and *y*-directions, respectively. *S_m_* represents a mass addition to the continuous phase, which is zero in this case. The first term of the left-hand side of the equation shows the local derivative which is physically the time rate of change of density at the fixed point. The second and third terms show the convective derivative of the density of the gas mixture [35].

Momentum equations in *x* and *y*-directions

(12) $$\begin{array}{c} \frac{\partial \left(\rho u_{x}\right)}{\partial t}+\frac{\partial \left(\rho u_{x}u_{x}\right)}{\partial x}+\frac{\partial (\rho u_{x}u_{y})}{\partial y}=-\frac{\partial P}{\partial x}+\frac{\partial }{\partial x}\left[\frac{4}{3}µ\frac{\partial u_{x}}{\partial x}-\frac{2}{3}µ\frac{\partial u_{y}}{\partial y}\right]\\ +\;\frac{\partial }{\partial y}\left[µ\left(\frac{\partial u_{y}}{\partial x}+\frac{\partial u_{x}}{\partial y}\right)\right] \end{array}$$

(13) $$\begin{array}{c} \frac{\partial \left(\rho u_{y}\right)}{\partial t}+\frac{\partial \left(\rho u_{y}u_{x}\right)}{\partial x}+\frac{\partial (\rho u_{y}u_{y})}{\partial y}=-\frac{\partial P}{\partial y}+\frac{\partial }{\partial y}\left[\frac{4}{3}µ\frac{\partial y}{\partial y}-\frac{2}{3}µ\frac{\partial u_{x}}{\partial x}\right]\\ +\frac{\partial }{\partial x}\left[µ\left(\frac{\partial u_{y}}{\partial x}+\frac{\partial u_{x}}{\partial y}\right)\right] \end{array}$$

where *P* represents the stream pressure and µ is the viscosity of the gas stream. The first term of the right-hand side of each equation shows pressure forces. The second and third terms of the right-hand side of each equation show the viscous forces [36].

Energy equation

(14) $$\rho \frac{de}{dt}=-P\;div\;u+div\;\left(k\;grad\;T\right)+\phi +S$$

(15) $$div\;u=\frac{\partial u_{x}}{\partial x}+\frac{\partial u_{y}}{\partial y}$$

(16) $$grad\;T=\frac{\partial T}{\partial x}+\frac{\partial T}{\partial y}$$

(17) $$\dot{\iota }=C_{v}T$$

(18) P=ρRT

(19) $$\phi =µ\left\{2\left[{\left(\frac{\partial u_{x}}{\partial x}\right)}^{2}+{\left(\frac{\partial u_{y}}{\partial y}\right)}^{2}\right]+{\left(\frac{\partial u}{\partial y}+\frac{\partial v}{\partial x}\right)}^{2}\right\}$$

where e is the specific internal energy, $C_{v}$ is specific heat constant, and *k* is the thermal conductivity. ϕ shows the rate of dissipation energy per unit volume and *s* denotes the work done per unit volume by body forces. The first term of the right-hand side of Equation (14) is the rate of work done per unit volume, and the second term is the rate of heat transfer per unit volume through conduction [37]. 

Species transport equation

(20) $$\frac{\partial \left(\rho Y_{i}\right)}{\partial t}+\frac{\partial \left(\rho u_{x}Y_{i}\right)}{\partial x}+\frac{\partial (\rho u_{y}Y_{i})}{\partial y}=-\left[\frac{\partial J_{i,x}}{\partial x}+\frac{\partial J_{i,y}}{\partial y}\right]+{\dot{S}}_{i}$$

(21) $$J_{i,x}=-\rho D_{i}\frac{\partial Y_{i}}{\partial x}$$

(22) $$J_{i,y}=-\rho D_{i}\frac{\partial Y_{i}}{\partial y}\;\;$$

where $Y_{i}$ is a mass fraction, $D_{i}$ is a diffusion coefficient, $J_{i}$ is a mass flux of component *i,* and ${\dot{S}}_{i}$ is the net production rate of species through chemical reactions [36].

### 3.4. Kinetic Models Describing the Surface Catalytic Reactions

For the steam reforming of methane over rhodium, the detailed heterogeneous reaction scheme proposed by Karakaya et al. is employed [38]. It involves 44 elementary reactions with 6 gaseous and 13 surface species. The rhodium catalyst having surface site density Γ_Rh_ = 2.72 × 10^−9^ mol/cm^2^ is used. The total molar production rate of species *i*-th by surface reactions is given by the following [39]:

(23) $$\dot{Si}=\sum_{n=1}^{n_{s}}v_{i,n}k_{f,r}\prod_{j=1}^{Ng+Ns}C_{j}{}^{{v^{\prime }}_{j,n}},$$

where $\dot{Si}$ is the overall rate expression of *i*-th species for the gas phase or surface phase, $n_{s}$ is the number of surface elementary reactions, $v_{i,n}$ and ${v^{\prime }}_{j,n}$ are the stoichiometric coefficients, and Ng and Ns are the number of gas-phase and surface-phase species, respectively. The concentrations $C_{j}$ of adsorbed species are given in mol/m^2^.

As binding states of the adsorption of all species vary with the surface coverages, the temperature dependence of the reaction rate coefficients is determined using the modified Arrhenius expression [40]:

(24) $$K_{f,r}=A_{kr}T^{\beta_{kr}}exp\left(-\frac{E_{a_{r}}}{RT}\right)\theta_{i}^{\mu_{ir}}exp\left[\frac{\varepsilon_{ir}\theta_{i}}{RT}\right]$$

where $k_{f,r}$ is a forward rate coefficient, $A_{kr}$ is the pre-exponential factor, $\beta_{kr}$ is the temperature exponent, $E_{a_{r}}$ is the activation energy of the reaction *r*, *θ_i_* is the surface coverage with adsorbed species, and coefficients $\mu_{ir}$ and $\varepsilon_{ir}$ describe the dependence of the rate coefficients on the surface coverage of *i*-th species. Sticking coefficients are commonly used for adsorption reactions and are converted to conventional rate coefficients according to [41]:

(25) $$K_{f,r}^{ads}=\frac{S_{i}^{o}}{\Gamma^{\tau }}\sqrt{\frac{RT}{2\pi M_{i}}}$$

where $K_{f,\;r}^{ads}$ are the reaction rate constant for adsorption reactions, $S_{i}^{o}$ is the initial sticking coefficient, Γ is the surface site density, *τ* is the number of sites occupied by the adsorbing species, and $M_{i}$ is the molar mass of *i*-th species.

### 3.5. Computational Schemes

**Figure 5:**
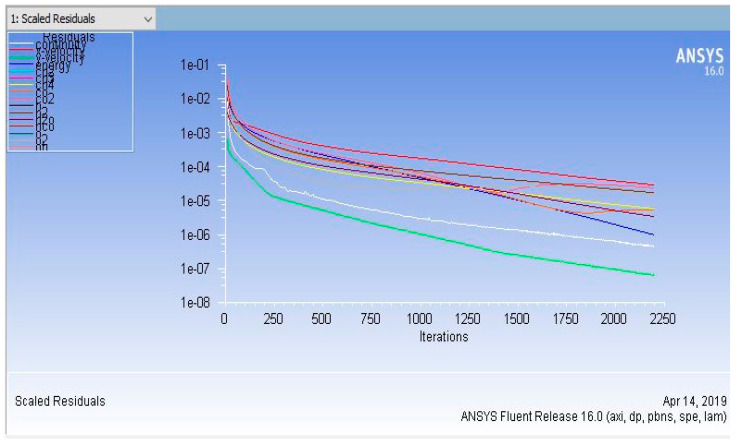
Convergence residual of our computational fluid dynamics (CFD) model.

The governing equations (i.e., momentum, energy, continuity, and species conservation) were discretized by using the finite volume method and were solved numerically by Fluent 16.0®. The second-order upwind scheme was used to discretize the mathematical model. The semi-implicit method for pressure-linked equations (SIMPLE) algorithm was used. Exergy analysis was performed by developing a custom field function (CFF)-based algorithm. An under-relaxation factor was used to slow down the rate of change. The default reference frame was used for velocity initialization. The Mach number, the ratio of flow velocity to the speed of sound, was used to determine whether the flow was compressible or incompressible. The Mach number was less than 0.3 in our model due to the low velocity of the gas stream; consequently, compressibility effects were ignored, and a pressure-based solver was used. Furthermore, the laminar finite rate model was used for calculating the rate of reactions. Computations are very intensive, and the convergence of CFD simulations was evaluated based on the residuals of all governing equations. The governing equations in our CFD model were converged at 2196, the number of iterations, as shown in Figure 5.

**Figure 6:**
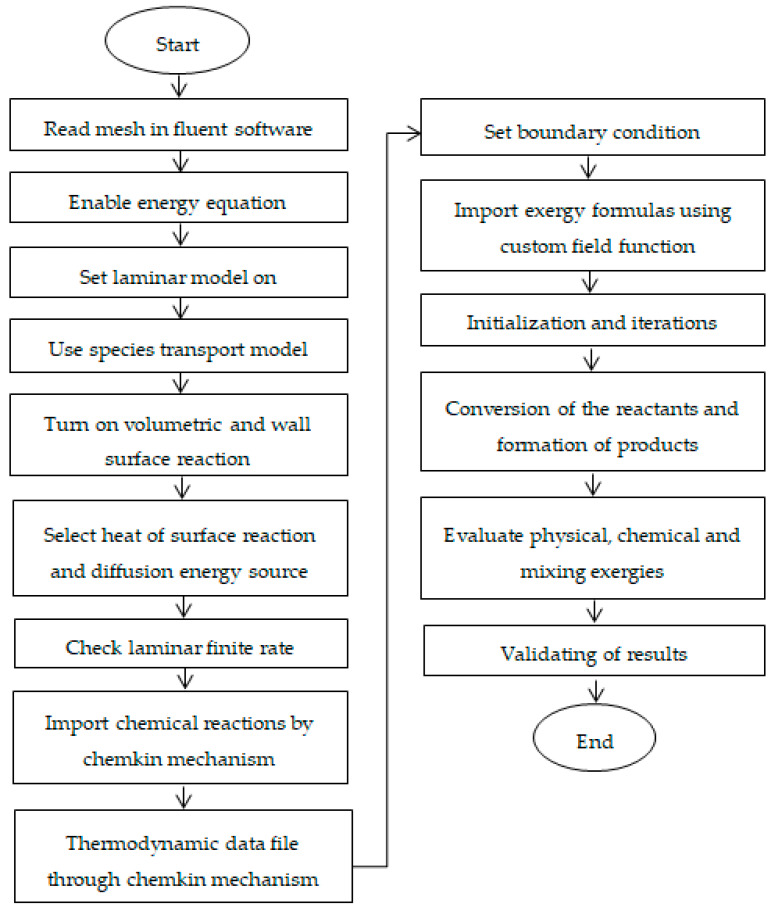
Schematic of model development process.

A schematic flowchart of the methodology adopted in this study is shown in Figure 6. Model development starts with geometry preceded by mesh preparation and identification of the boundary and zone cell conditions. Then, the ANSYS Fluent simulator reads the mesh and its properties. As temperature variations occur due to reaction kinetics, the energy equation was enabled in ANSYS Fluent to analyze the effect of temperature. Reaction kinetics and reaction thermodynamics were imported through the CHEMKIN file. The formulas for physical, chemical, mixing, and total exergy were generated and imported to the ANSYS Fluent software through the CFF. Changes in a mole fraction of species and exergy profiles were evaluated.

## 4. Results and Discussion

**Figure 7:**
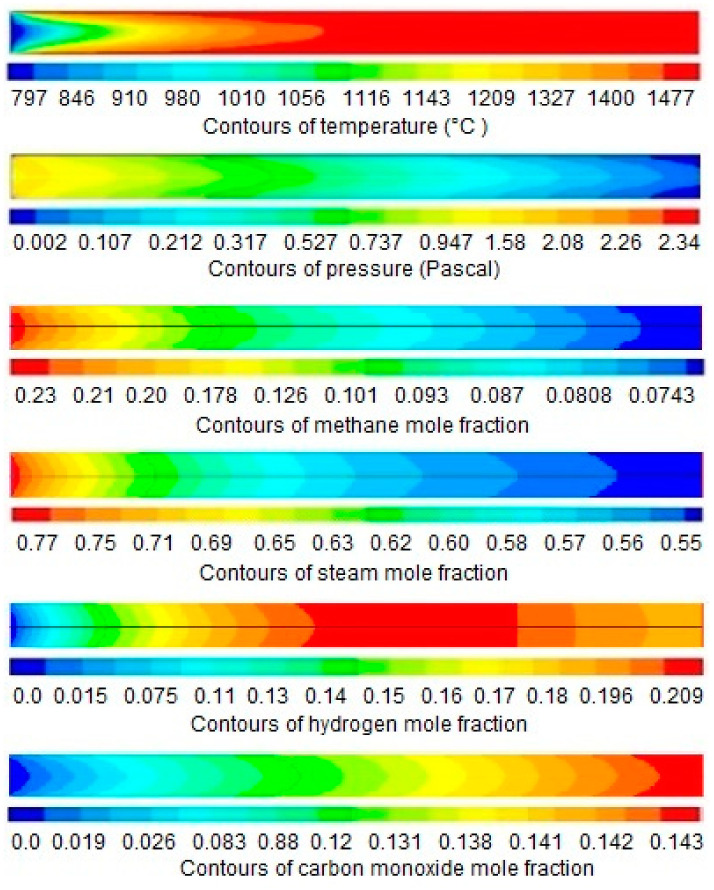
Total contours of simulation results along the length of the reactor (0–14 mm).

**Figure 8:**
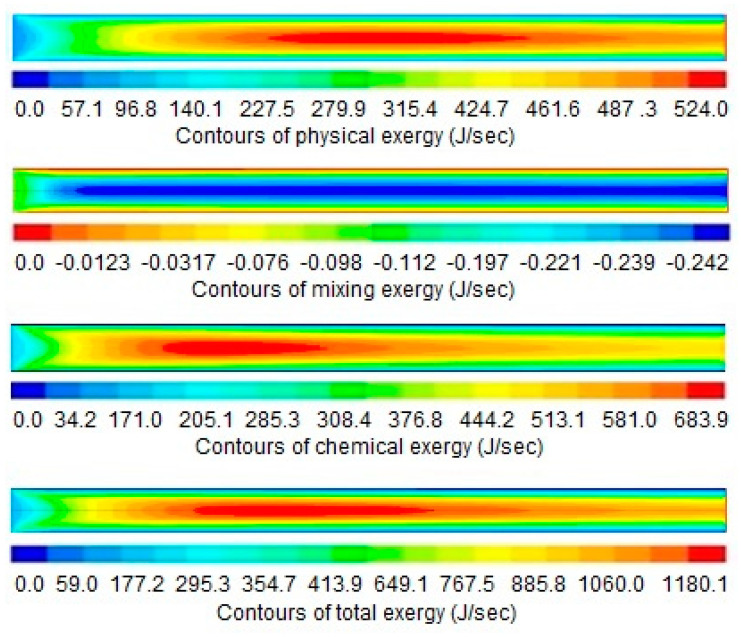
Contours of three types of exergies and total exergy, along the length of the reactor (0–14 mm).

**Figure 9:**
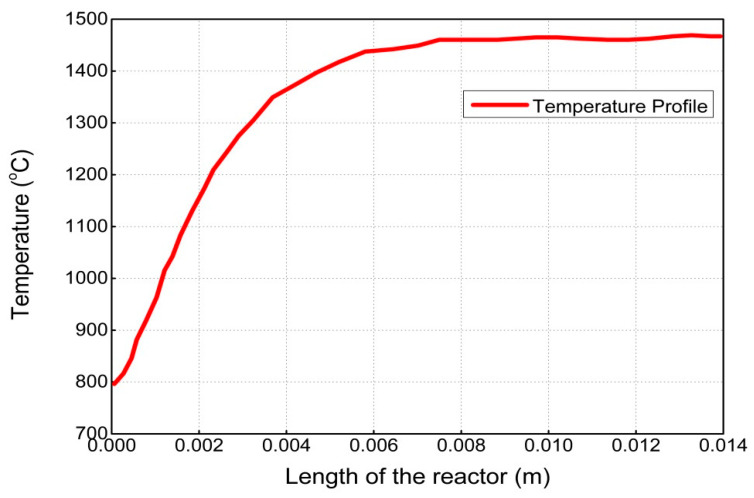
Temperature profile along the length of the reactor.

**Figure 10:**
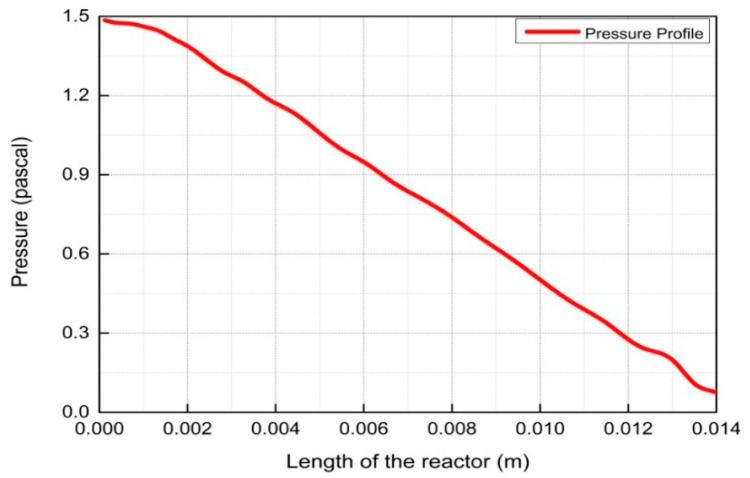
Pressure profile along the length of the reactor.

**Figure 11:**
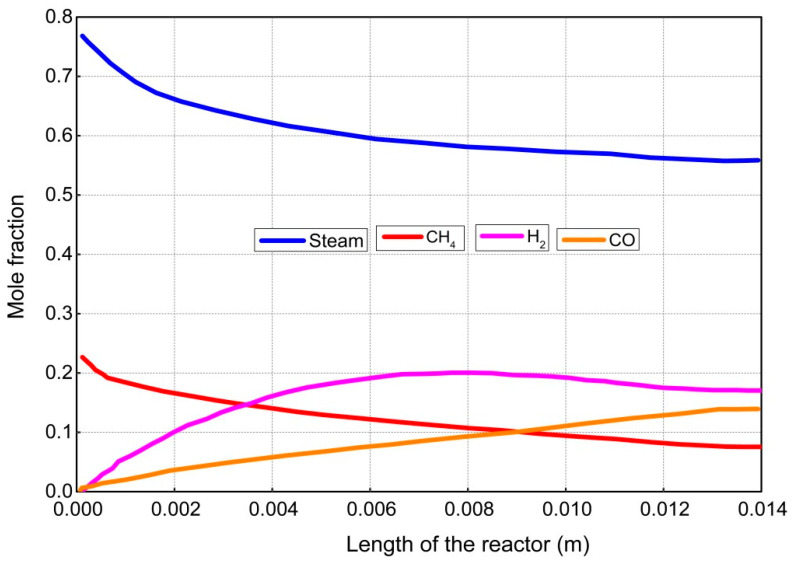
Conversion profiles of the reactants and formation of the products along the length of the reactor.

**Figure 12:**
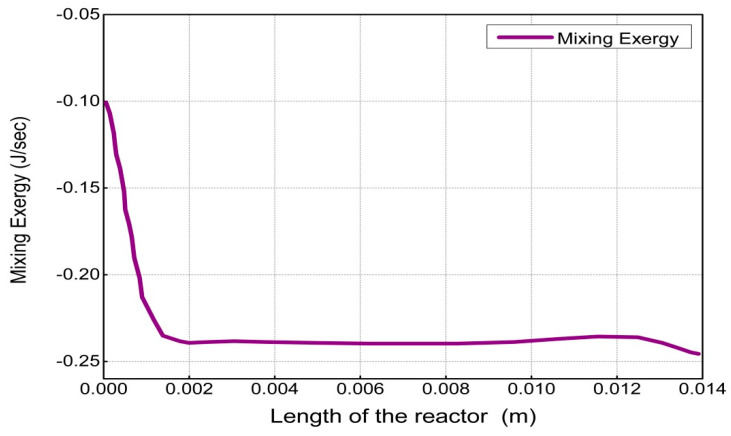
Mixing exergy profile along the length of the reactor.

**Figure 13:**
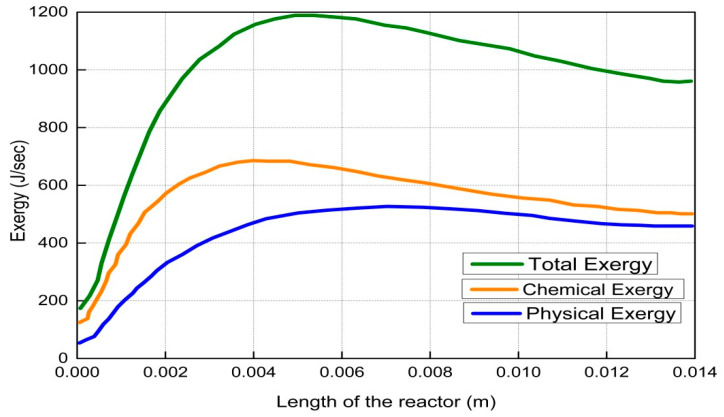
Profiles of two types of exergies and total exergy along the length of the reactor.

The results and discussion encompass contours and profiles of temperature, pressure, mole fractions of reactants and products, and exergy profiles of the reactor model. Contours of temperature, pressure, mole fractions of reactants, and products are shown in Figure 7, whereas the contours of three types of exergy and total exergy are shown in Figure 8. The profiles of temperature and pressure are shown in Figure 9 and Figure 10, respectively, whereas the mole fractions of methane, steam, hydrogen, and carbon monoxide are shown in Figure 11. The mixing exergy profile is demonstrated in Figure 12, followed by the physical, chemical, and total exergy profiles in Figure 13.

The temperature contours and profile shown in Figure 7 and Figure 9, respectively, portray the change in temperature in the longitudinal direction. A constant amount of heat is supplied through the wall of the reactor where the temperature drops in the first part of the reactor (i.e., up to 8 mm) due to the consumption of heat in an endothermic reaction. On the completion of the reactions, in the later part of the reactor (i.e., up to 14 mm), less amount of heat is consumed, and the temperature remains high and constant.

Figure 7 and Figure 10 show the pressure contours and their graph, respectively, revealing the decreasing trend linearly along the length of the reactor. The continuous pressure drop is due to the increase of fluid velocity along the length of the reactor. The increase in velocity causes the pressure drops inversely in fluids. The same effect is stated by the Bernoulli equation.

The contours and the graph of methane conversion are shown in Figure 7 and Figure 11, respectively, portraying that the mole fraction is decreasing rapidly from 0.23 to 0.075 (i.e., up to 12 mm), then remains constant. Methane, a limiting reactant, is converted up to 67.4%. In a previous study reported in the literature, 60% conversion of methane was achieved in a monolith reactor. The conversion of excess reactant (i.e., steam) is shown in Figure 7 and Figure 11, respectively, where the mole fraction of steam drops from 0.77 to 0.56.

The formation of hydrogen is shown in Figure 7 and Figure 11, respectively, demonstrating that the concentration of hydrogen increases along the length of the reactor up to 9 mm. A slight decrease was then noticed up to 12 mm. The decrease in concentration may be caused by the formation of intermediates and reversible reactions, as shown in Table 3. Similarly, the concentration profile of carbon monoxide is shown in Figure 7 and Figure 11, respectively, where the concentration of carbon monoxide is continuously increasing along the length of the reactor.

The performance of the proposed model is compared with a model reported by Cao et al. in the literature [36]; see Table 4. The proposed model achieved a 7.4% higher conversion with 76.6 % smaller surface area compared to the reported work [36]. The higher conversion at the shorter reactor is due to a higher wall temperature, namely, 1477 °C), in the proposed work compared to 900 °C in the reported work. However, total heat consumption in the proposed model is 65.0% lower than the reported work due to the smaller surface area. Furthermore, the reduction in the size of the reactor resulted in a 76.7% lesser requirement of the catalyst in comparison with the reported work [36].

The mixing exergy always has a negative value as exergy of pure components is higher than the components in the mixed form [28]. Decrease in total exergy due to mixing is demonstrated in Figure 8 and Figure 12, respectively. The high conversion rate in the reactor produces new product species at a faster rate. The high-speed molecules intensify the mixing effects. These effects create major irreversibility and contribute significantly to the overall exergy destruction in the reactor. This trend of mixing exergy is validated by another study reported in the literature [17].

The contour and profile of physical exergy are shown in Figure 8 and Figure 13, respectively. At the start of the reactor, the quantity of physical exergy is low which then increases rapidly up to 7 mm. The low quantity of physical exergy at the start of the reactor is due to the low temperature as shown in Figure 7 and Figure 9, respectively. The low temperature is due to the consumption of heat by endothermic reactions of steam methane reforming. The temperature drop causes irreversibility, which results in a decrease in physical exergy at the start of the reactor. A slight decrease in the physical exergy from 7 mm to 12 mm is observed, which is caused by the continued decrease of pressure from start to end of the reactor; physical exergy at this portion only depends on the pressure because its temperature remains constant.

The contours and profile of chemical exergy increase from left to right of the reactor as shown in Figure 8 and Figure 13, respectively. Chemical reactions take place in the catalytic bed and produce new species. These species have high chemical potential which increases the total chemical exergy.

Total exergy is the summation of physical, mixing, and chemical exergy, which is shown in Figure 8 and Figure 13, respectively. The total exergy increases from the start of the reactor till the end of the reactor. The increase in total exergy is due to the combined increasing effect of physical and chemical exergies. The increase in total exergy results in higher work potential of the product, syngas.

## 5. Conclusions

In this work, the computational fluid dynamics (CFD)-based method was adopted to perform an exergy analysis of the monolith microreactor of the steam methane reforming (SMR) process. Initially, the CFD model of SMR was developed using literature data. In order to incorporate reaction kinetics, CHEMKIN was used. By optimizing the size and the operation conditions, the optimal SMR microreactor achieved a 7.4% higher conversion with 76.6 % smaller surface area compared to the reported work. The higher conversion achieved by the shorter reactor is due to a higher wall temperature, namely, 1477 °C, in the proposed work compared to 900 °C in the reported work. Although the temperature used in the proposed work is higher than the reported work, total heat consumption in the proposed work is 65.0% lower due to the smaller surface area. Furthermore, the reduction in the size of the reactor resulted in a 76.7% reduction in the catalyst requirement. 

The exergy analysis was performed by developing a custom field function (CFF)-based algorithm. The exergy analysis helped in evaluating length-wise profiles of all three types of exergy, namely, physical exergy, chemical exergy, and mixing exergy, in the microreactor. The results show that the physical and chemical exergies increase due to the increase in temperature and high chemical potential of product species, respectively. On the other hand, the mixing exergy decreases due to the high rate of mixing effects that causes irreversibility. 

In future work, sensitivity analysis and uncertainty analysis will be performed to achieve a further optimization of the process conditions. The sensitivity analysis helps in evaluating the individual impact of process conditions on its outcome, whereas the uncertainty analysis is used to quantify the collective impact of variation in process conditions on its outcome.

## Figures and Tables

**Table 1:** Model parameters.

*Parameters*	*Symbol*	*Value*
Channel length	L	14 mm
Channel diameter	D	1 mm
Inlet temperature	T_in_	800 °C
Isothermal wall temperature	T_w_	1477 °C
Pressure	P	1 atm
Ratio of steam to methane	S/C	3:1
Inlet velocity	V_in_	0.45m/s
Number of catalyst active sites (catalyst density)	Γ	2.7 × 10^−9^ mol/cm^2^

**Table 2:** Mesh properties.

*Properties of Mesh*	*Values*
Orthogonal quality (minimum)	1.0
Ortho skew (maximum)	0.0
Aspect ratio (maximum)	1.4
The number of nodes	7525
The number of cells	7200
Minimum volume (m^3^)	2.4 × 10^−13^
Maximum volume (m^3^)	2.3 × 10^−11^
Total volume (m^3^)	4.2 × 10^−8^
Minimum face area (m^2^)	4.1 × 10^−5^
Maximum face area (m^2^)	4.5 × 10^−5^

**Table 3:** Elementary reactions and their kinetics for steam methane reforming on the Rh catalyst [33].

*Serial No.*	*Reactions*	*A_kr_ (mol, cm, s, K)*	$S_{i}^{o}{\left(-\right)}^{\;}$	*E_ar_ [KJ/mol]*
1.	H_2_ + 2Rh(s)→2H(s)		0.01	0.0
2.	2H(s)→H_2_ + 2Rh(s)	3.0 × 10^21^		77.8
3.	O_2_ + 2Rh(s)→2O(s)			
4.	2O(s)→O_2_ + 2Rh(s)	1.33 × 10^22^		355.2
5.	CH_4_ + Rh(s)→CH_4_(s)		8.0 × 10^−3^	0.0
6.	CH_4_(s)→CH_4_ + Rh(s)	2.0 × 10^14^		25.1
7.	H_2_O + Rh(s)→H_2_O(s)		0.1	0.0
8.	H_2_O(s)→H_2_O + Rh(s)	6.0 × 10^13^		45
9.	CO_2_ + Rh(s)→CO_2_(s)		1.0 × 10^−5^	0.0
10.	CO_2_(s)→CO_2_ + Rh(s)	3.0 × 10^8^		21.7
11.	CO + Rh(s)→CO(s)		5 × 10^−1^	0.0
12.	CO(s)→CO + Rh(s)	1.0 × 10^13^		133.4
13.	H(s) + O(s)→OH(s) + Rh(s)	5.0 × 10^22^		83.7
14.	OH(s) + Rh(s)→ H(s) + O(s)	3.0 × 10^20^		37.7
15.	H(s) + OH(s)→H_2_O(s) + Rh(s)	3.0 × 10^20^		33.5
16.	H_2_O(s) + Rh(s)→ H(s) + OH(s)	5.0 × 10^22^		106.4
17.	2OH(s)→H_2_O(s) + O(s)	3.0 × 10^21^		100.8
18.	H_2_O(s) + O(s)→ 2OH(s)	3.0 × 10^21^		171.8
19.	C(s) + O(s)→CO(s) + Rh(s)	5.0 × 10^23^		97.9
20.	CO(s) + Rh(s)→C(s) + O(s)	3.7 × 10^21^		169.0
21.	CO(s) + O(s)→CO_2_(s) + Rh(s)	1.0 × 10^19^		121.6
22.	CO_2_ + Rh(s)→ CO(s) + O(s)	5.0 × 10^21^		115.3
23.	CO(s) + H(s)→HCO(s) + Rh(s)	5.0 × 10^19^		108.9
24.	HCO(s) + Rh(s)→ CO(s) + H(s)	3.7 × 10^21^		0.0
25.	HCO(s) + Rh(s)→CH(s) + O(s)	8.0 × 10^23^		59.5
26.	CH(s) + O(s)→ HCO(s) + Rh(s)	3.7 × 10^21^		167.5
27.	CH_4_(s) + Rh(s)→CH_3_ + H(s)	5.5 × 10^20^		61.0
28.	CH_3_ + H(s)→ CH_4_(s) + Rh(s)	3.7 × 10^21^		51.0
29.	CH_3_(s) + Rh(s)→CH_2_(s) + H(s)	3.7 × 10^21^		103.0
30.	CH_2_(s) + H(s)→ CH_3_(s) + Rh(s)	3.7 × 10^21^		44.0
31.	CH_2_(s) + Rh(s)→CH(s) + Rh(s)	3.7 × 10^34^		100.0
32.	CH(s) + Rh(s)→ CH_2_(s) + Rh(s)	3.7 × 10^34^		68.0
33.	CH(s) + Rh(s)→C(s) + H(s)	3.7 × 10^21^		21.0
34.	C(s) + H(s)→ CH(s) + Rh(s)	3.7 × 10^21^		172.8
35.	CH_4_(s) + O(s)→CH_3_(s) + OH(s)	1.7 × 10^24^		80.3
36.	CH_3_(s) + OH(s)→CH_4_(s) + O(s)	3.7 × 10^21^		24.3
37.	CH_3_(s) + O(s)→CH_2_(s) + OH(s)	3.7 × 10^24^		120.3
38.	CH_2_(s) + OH(s)→CH_3_(s) + O(s)	3.7 × 10^21^		15.1
39.	CH_2_(s) + O(s)→CH(s) + OH(s)	3.7 × 10^24^		114.5
40.	CH(s) + OH(s)→CH_2_(s) + O(s)	3.7 × 10^21^		36.8
41.	CH(s) + O(s)→C(s) + OH(s)	3.7 × 10^21^		30.1
42.	C(s) + OH(s)→CH(s) + O(s)	3.7 × 10^21^		136.0
43.	CO(s) + H(s)→C(s) + OH(s)	3.7 × 10^21^		142.0
44.	C(s) + OH(s)→CO(s) + H(s)	3.7 × 10^20^		25.5

**Table 4:** Comparison of the proposed model with the model reported in the literature [36].

Parameters	Literature Data [36]	Proposed Model Data
Feed temperature	800 °C	800 °C
Wall temperature	900 °C	1477 °C
Pressure	1 atm	1 atm
Steam to methane ratio	3:1	3:1
Inlet velocity	0.45 m/s	0.45m/s
Length	6.0 × 10^−2^ m	1.4 × 10^−2^ m
Surface area	1.88 × 10^−4^ m^2^	4.4 × 10^−5^ m^2^
Heat requirement	22.05 kW	7.7 kW
Conversion	60 %	67.4 %
Catalyst requirement per length	5.076 × 10^−9^ moles	1.18 × 10^−9^ moles

## Abbreviations

CFD	computational fluid dynamics
SMR	steam methane reforming
PSA	pressure swing adsorption
E^ph^	molar physical exergy
E^mix^	molar mixing exergy
E^ch^	molar chemical exergy
${\bar{G}}_{i}$	molar Gibbs function
R	Ideal gas constant
$C_{v}$	specific heat constant
ϕ	dissipation energy per unit volume
$Y_{i}$	mass fraction
$D_{i}$	diffusion coefficient
$J_{i}$	mass flux of component i
${\dot{S}}_{i}$	production rate
$k_{f,r}$	forward rate coefficient
$A_{kr}$	pre-exponential factor of the reaction r
$\beta_{k}$	temperature exponent
$E_{a_{r}}$	activation energy of the reaction r
Γ	surface site density
$M_{i}$	molar mass of *i*-th species
Rh	Rhodium catalyst
